# Proportion of Mental Defectives among Delinquents

**Published:** 1915-11

**Authors:** 


**Proportion of Mental Defectives Among Delinquents.** Augusta F. Bronner of the Psychopathic Institute of the Juvenile Court of Chicago, Jour. of Am. Inst. of Crim. Law and Criminology, Nov. 1914, considers that the estimate of nearly 90% of mental deficiency in juvenile offenders is too high. She holds that such percentages are due to using only the caught—and therefore less intelligent—delinquents, as a basis of statistics, to too literal reliance on the Binet test, to inability to understand language and to the personal equation of the examiner. She considers that, of her material, more than 90% are intellectually normal. (Note: It is very difficult to eliminate the personal equation and possibly Miss Bronner is prejudiced, like the reviewer, against attributing crime to mental deficiency for which the offender is not responsible. Common sense and accumulated experience must ultimately settle the true balance between the extremes of punishing delinquents for acts for which they are not responsible and of advocating the reduction of our criminal laws and courts to the basis of regulation of therapeutic measures for some mental disease).

